# FluoroMatch IM: An Interactive Software for PFAS Analysis by Ion Mobility Spectrometry

**DOI:** 10.1021/acs.est.4c13846

**Published:** 2025-03-25

**Authors:** Rachel Smolinski, Jeremy P. Koelmel, Paul Stelben, David Weil, David Godri, David Schiessel, Michael Kummer, Sarah M. Stow, Sheher Mohsin, Lauren Royer, Alan McKenzie-Coe, Thomas Lubinsky, Daniel DeBord, Olivier Chevallier, Emma E. Rennie, Krystal J. Godri Pollitt, Carrie McDonough

**Affiliations:** † Department of Chemistry, Carnegie Mellon University, Pittsburgh, Pennsylvania 15213, United States; ‡ Department of Environmental Health Science, Yale School of Public Health, New Haven, Connecticut 06520, United States; § Agilent Technologies, Inc., Santa Clara, California 95051, United States; ∥ third Floor Solutions, Toronto, ON 43964, CA; ⊥ Innovative Omics, Inc., Sarasota, Florida 34235, United States; # MOBILion Systems, Inc. Chadds Ford, Chadds Ford, Pennsylvania 19317, United States

**Keywords:** PFAS, ion mobility, software, nontarget analysis, HRMS

## Abstract

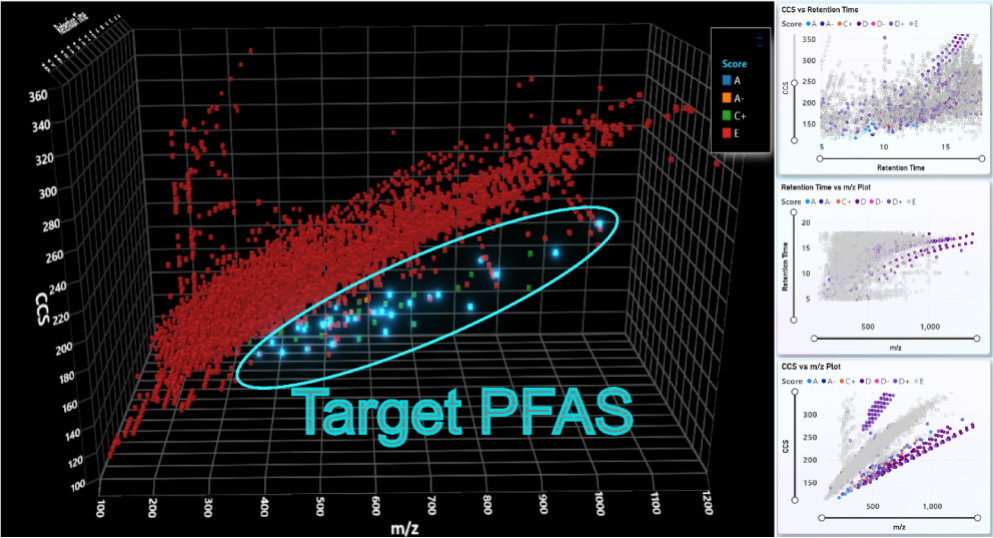

Per- and polyfluoroalkyl substances (PFASs) are often present in complex mixtures at trace levels in environmental samples, posing difficulties for analytical chemists. Ion mobility offers highly replicable identifiers, enabling the use of community-based libraries for PFAS annotation in nontargeted analysis. Currently, limited software exists to leverage the capabilities of liquid chromatography ion mobility high-resolution mass spectrometry (LC-IM-HRMS) for nontargeted analysis. FluoroMatch IM is a free vendor-neutral open-source tool for rapid annotation of PFASs in LC-IM-HRMS datasets. Annotation algorithms include collision cross-section (CCS) matching, formula prediction, homologous series detection, mass defect filtering, and accurate mass matching with a database of 194 PFAS ions that can be continuously expanded by the community. Results from FluoroMatch IM were compared to a targeted approach with a laboratory-prepared mixture of 63 PFASs and real wastewater samples. A nontarget workflow incorporating FluoroMatch IM revealed additional likely PFASs (*n* = 16) while confirming most targeted annotations (11/12) in wastewater samples. Validation of the standard mix showed a low false negative rate of 5% and a 5% false positive rate for features included in the CCS library, with a 0% false positive rate for features assigned confident scores. This study demonstrates the promise of FluoroMatch IM for streamlining PFAS analysis workflows.

## Abstract 2

FluoroMatch IM enables rapid annotation of untargeted ion mobility data for PFASs in complex environmental and biological samples.

## 1 Introduction

Per-/polyfluoroalkyl substances (PFASs) are anthropogenic chemicals that are ubiquitous in the environment, defined by the OECD as having at least one fully fluorinated carbon moiety.^1^ Detection and identification of PFASs in complex matrices present challenges, as there are thousands of identified PFASs, many of which lack analytical standards.^2,3^ Improved resolution in mass spectrometry has allowed for the tentative identification of novel exogenous chemicals without the need for reference standards; however, high-resolution mass spectrometry (HRMS) alone does not eliminate all the challenges in PFAS identification. Comprehensive detection of PFASs in environmental and biological samples requires sophisticated algorithms to compile all pieces of evidence for chemical assignments, expert review of the results, and consensus on reporting and quality control guidelines.

With tens of thousands of chromatographic features in HRMS data, there is a marked interest in prioritizing features that are likely PFASs to minimize the extensive manual data verification. Numerous techniques have been developed to prioritize PFASs in complex matrices, including suspect screening, Kendrick mass defect analysis,^4,5^ mass defect filtering, homologous series identification,^6,7^ screening for common PFAS fragments,^8^ and applying *m*/*z*-retention time filtering.^9^ These techniques each offer different advantages in data prioritization and structural confirmation. Of these techniques, fragmentation evidence is often key for structure annotation, with a <5% false positive rate achievable using class-based patterns and fragmentation rules.^7,10−13^ Homologous series are determined using repeating mass intervals (e.g., 50 Da for CF_2_) and normalized mass defect (e.g., normalized to CF_2_). Once annotations are determined using fragmentation rules, homologous series detection can reveal additional homologues within the same PFAS subclass differing in the number of repeating units (e.g., CF_2_). Techniques leveraging accurate mass alone (e.g., homologous series detection, Kendrick mass defect and mass defect filtering, accurate mass matching) will always have high false positive rates due to the percent overlap between molecules in accurate mass space. For example, using the EPA CompTox Dashboard PFAS^14^ list from 2019, 37% of PFASs had an overlapping accurate mass within 0.005 Da, necessitating the need for fragmentation or other orthogonal lines of evidence to confirm molecular structures. Albeit powerful, the use of fragment-based annotations and homologous series is limited. The number of annotations, and hence the false negative rate, will always be limited to homologous series with quality MS/MS spectra for at least one feature and overlooks PFASs that may not belong to a series. In the case of samples with trace levels of PFASs, signals are often too low to produce quality MS/MS spectra.^13,15^ Therefore, an orthogonal line of evidence is desired for challenging trace-level annotations.

Ion mobility spectrometry (IMS) provides a method of separation in which molecules are separated by charge state, size, and molecular configuration, allowing for measurements independent of chromatography and mass spectrometry techniques. Drift tube IMS (DTIMS) instruments allow for the calculation of collision cross section (CCS) values from measured ion drift times and show high reproducibility among DTIMS instruments, typically within 2% in interlaboratory studies,^16^ thus providing a dependable identifier beyond exact mass.^16,17^ While IMS instruments differ in ion transmission principles and measurements, collision cross section (CCS) values are still highly comparable between instrument platforms.^16,17^ This allows the use of community-based libraries to aid in annotation for PFAS analysis.^18−20^ The publication and applied use of publicly available CCS libraries is a growing field and has shown promise in increasing confidence in annotations of lipids, PFASs, and other organic micropollutants in complex samples.^18,21−23^ Applications of IMS in the PFAS space have facilitated the separation of isomers previously undifferentiated by chromatography and MS/MS, and researchers have leveraged distinct mass-CCS trendlines of PFAS subclasses to improve sample characterization and identify members of homologous series.^19^ PFASs are also known to exhibit relatively lower CCS values at a given *m*/*z* when compared to endogenous molecules due to the relatively higher weight (but not comparably greater atomic radius) of fluorine versus hydrogen, providing an effective approach to differentiating fluorinated chemicals from biological space.^18^ IMS separation is also leveraged to produce filtered fragmentation (MS/MS) spectra as fragmentation occurs after the drift cell; therefore, fragments are mobility-aligned with their respective precursor, improving spectral annotation and structure elucidation.^16^

This study aims to address analytical challenges posed by PFAS annotation by demonstrating FluoroMatch IM, a vendor-neutral tool that aids in nontargeted PFAS analysis using liquid chromatography ion mobility high-resolution mass spectrometry (LC-IM-HRMS). FluoroMatch IM leverages CCS values, drift time trends, and homologous series detection to facilitate the identification of PFASs. FluoroMatch IM detects homologous series using accurate mass, retention time order, drift time order, extracted ion chromatogram (EIC) visualization (in both drift time and retention time dimensions), mass spectra, formula prediction, and CCS matching using a built-in CCS library, which will be expanded over time by the user community.^24^

In this study, we evaluated FluoroMatch IM for the analysis of a laboratory-prepared mixture of 63 PFASs to determine false positive and false negative rates. To evaluate the application of FluoroMatch IM to PFAS characterization in real environmental mixtures, we then analyzed extracts from passive sampling of residential wastewater using traditional targeted approaches and FluoroMatch IM.

## 2 Methods

### 2.1 Software Algorithms and Analytical Workflow

**Figure 1:**
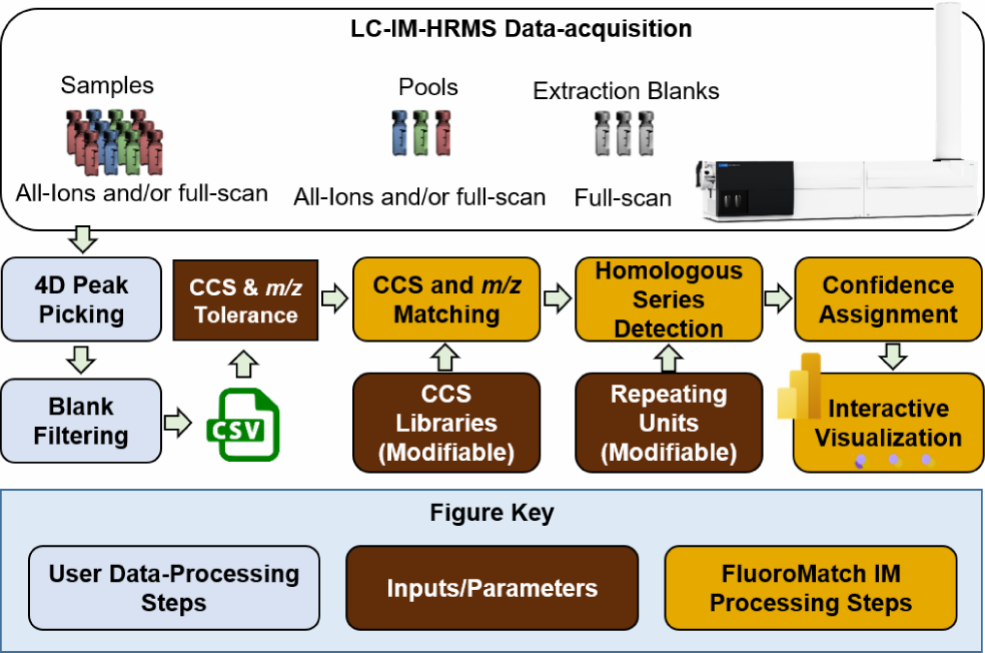
User workflow and FluoroMatch IM high-level algorithm workflow. Users first acquire (at a minimum) full scan data files and extraction blanks. They then perform their own 4D peak picking, choose tolerance, and then run FluoroMatch IM. 4D peak picking is used to determine features (defined by *m*/*z*, drift time, retention time, and ion intensity) and align them across samples. FluoroMatch IM performs accurate mass and CCS matching, homologous series detection, confidence assignment, and visualization.

All data-processing algorithms for FluoroMatch IM software were written in R v4.4^25^ and all visualizations are created in Microsoft Power BI (Desktop Version). The user workflow (Figure 1) consists of three steps: processing LC-IM-HRMS data to obtain a feature table, uploading the feature table with the correct parameters into the R script, and uploading the results into the Power BI visualizer. A feature-finding software of the user’s choice can be used to generate the feature table (feature-finding is not included with FluoroMatch IM), provided that the necessary information can be generated from acquired IMS-MS data: retention time, adduct mass-to-charge (*m*/*z*) (e.g., [M–H]^−^, [M–H–CO_2_]^−^), drift time, and CCS values. Any other columns included will be carried through all data-processing steps and can be used as factors for visualization (e.g., size by molecular abundance). A pop-up interface using the tcltk (Tool Command Language/ToolKit) R package is generated, where the user uploads the feature table, library file, and repeating units file.

A default CCS library is provided with FluoroMatch IM, containing CCS values for 194 PFAS ions generated from 99 PFASs and mass-labeled standards. CCS library entries were compiled from publicly available databases^18,26^ or measured by Agilent Technologies, and all library values were acquired using DTIMS (Agilent 6560) with nitrogen carrier gas. The ^DT^CCS_N2_ values in this library are anticipated to be consistent with CCS values generated from other instruments with calibration-calculated CCS values, such as traveling wave IMS (TWIMS) and cyclic IMS (cIMS) systems (see Section 3.1). The library file consists of molecule names, identifiers, molecular formulas, SMILES notation, adduct *m*/*z*, and CCS values, and it can be readily expanded by users to incorporate their own CCS entries. At a minimum, names, molecular formulas, adduct *m/z’s*, and CCS values are required. This library contains multiple adducts and common neutral losses, such as [M-H–CO_2_]^−^ of perfluoroalkyl carboxylates (PFCAs). The repeating units file contains repeating units that can be used to automatically detect homologous series, including CF_2_, CF_2_CF_2_O, CH_2_CF_2_, CH_2_CHF, CH_2_CH_2_CF_2_CF_2_, CF_2_CFCl, CH_2_CH_2_CF_2_CFCl, and OCF_2_CFCF_3_. Series can be toggled between TRUE and FALSE, and any number of series can be added. By default, only CF_2_ is set to TRUE, and hence only CF_2_ repeating unit series will be detected. While other series do exist and querying them can be helpful to enhance coverage, often the inclusion of many other series with larger repeating units leads to false positives and increased complexity during the manual review process.

**Figure 2:**
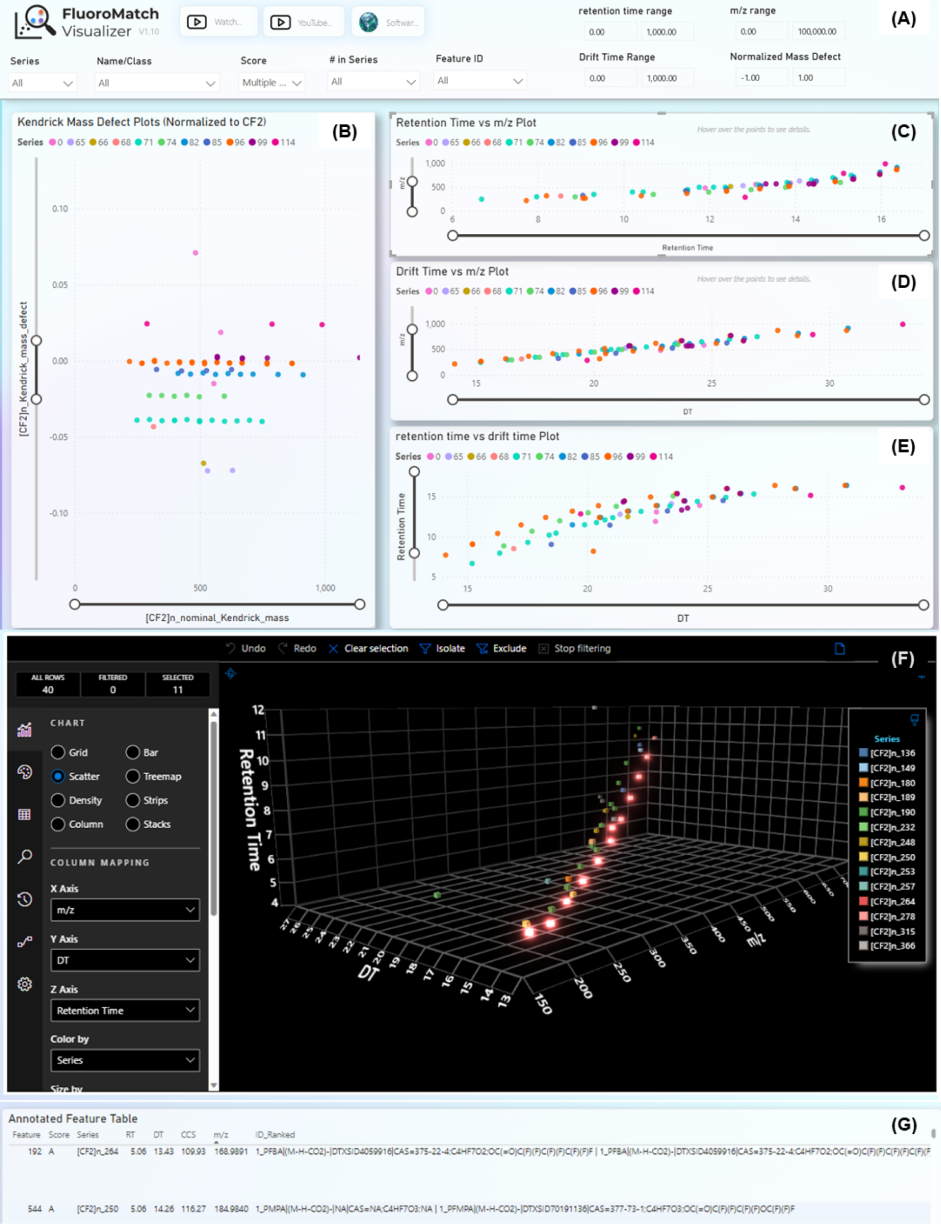
FluoroMatch IMVisualization framework, with all visualizations interactively filtered on selection and filtered via confidence assignment, series, number in series (after flagging), chemical class, retention time, drift time, and *m*/*z* range (shown in panel A). In addition, the following visualizations are shown; normalized mass defect plots (B), retention time versus *m*/*z* plots (C), drift time versus *m*/*z* plots (D), retention time versus drift time plots (E), a 3D plot of *m*/*z* versus drift time versus retention time (F), and an annotated feature table (G). All logos and icons are from Innovative Omics (innovativeomics.com).

Parameters that can be toggled by the user include precursor *m*/*z* tolerance (Da), CCS match tolerance window (%), mass defect range, and whether to include retention time and drift time order flagging. If drift time and retention time flagging are set to “Yes,” then members of series that do not follow an increasing value for both drift time and retention time with *m*/*z* are flagged and do not count toward that series total number of homologs. Note these flagged features are still categorized within the series they were assigned by accurate mass. After running the software, a .csv file ending in “FIN” will be generated, which can be uploaded into the distributed Power BI file. The user can then manually validate annotations and discover new annotations in the Visualizer (Figure 2).

**Figure 3:**
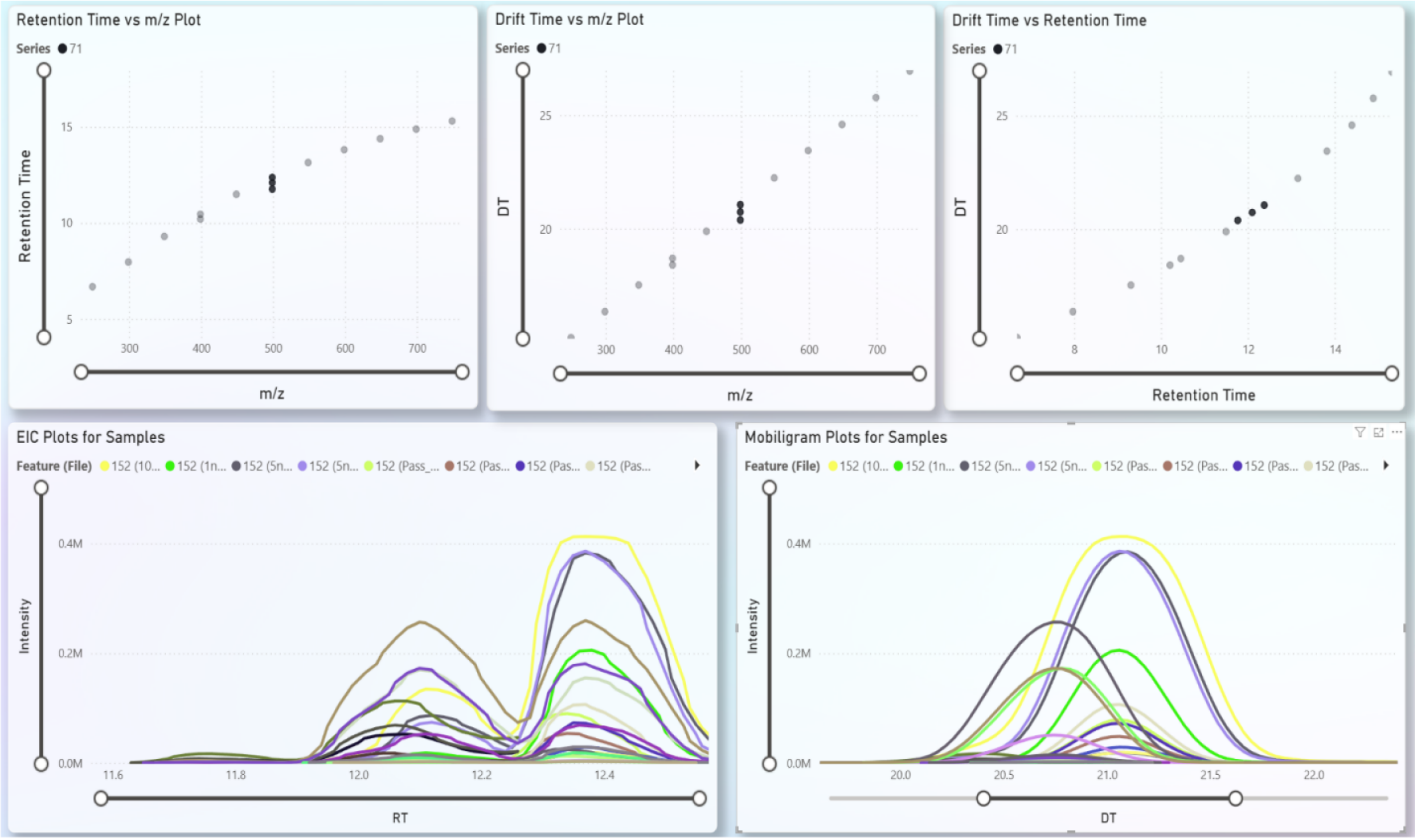
FluoroMatch IM Visualization framework with retention time versus *m*/*z*, drift time versus *m*/*z*, and drift time versus retention time for the perfluorosulfonic acid (PFSA) series (upper). EIC plots and mobilogram plots are shown for PFOS isomers (lower).

Visualizations include Kendrick mass defect plots normalized to a repeating unit (CF_2_ by default), extracted ion chromatograms, annotated MS1 spectra, experimental versus theoretical MS1 spectral isotopic patterns for formula matches, retention time versus *m*/*z* plots, drift time versus *m*/*z* plots, drift time versus retention time plots, and 3D plots of drift time versus retention time versus *m*/*z* (Figures 2 and 3). The data are color-coded by homologous series (Figure 2) and can be filtered by homologous series (Figure 3), which automatically pulls out the series detected by the FluoroMatch IM algorithm. Results can also be filtered by PFAS subclass (e.g., PFCAs), number of features in a series, confidence score, retention time range, drift time range, *m*/*z* range, normalized mass defect, and isotopic ratios to facilitate feature prioritization. Filtering the data by selecting specific features with the cursor automatically filters all plots.

The entire workflow described above is vendor-neutral and works for all vendor files, provided a 4-D feature table in .csv format can be obtained. Additional functionality (not demonstrated here) allows for integration of the Agilent LC-IM-HRMS/MS system All Ions MS/MS data. Briefly, the PNNL Preprocessor is used to generate files similar to data-dependent acquisition (DDA) files from LC-IM-HRMS/MS All Ions data; these files are generated by filtering All Ions MS/MS data to just a single scan based on each feature’s drift time and retention time. Hence, any MS/MS fragments from other ions fragmented across the entire mass range that fall within the same retention time and drift time will also be cofragmented (the chance of chimeric spectra is increased as compared to traditional data-dependent analysis). The resulting data-dependent formatted files are then imported into the FluoroMatch IM software, which contains all features described for FluoroMatch LC (Section S1).^7,11,24,27^ Briefly, in addition to the features mentioned in this manuscript, the MS/MS is leveraged to perform fragment screening (assigning fragments using a list of 777 PFAS-related fragments and corresponding substructures), PFAS subclass rule-based annotation (screening across over 15,000 species), and matching to *in silico* MS/MS spectra (screening across hundreds of thousands of PFAS, including predicted biotransformation products).^7,11,24,27^ The PFAS rule-based matching requires specific fragment matches (e.g., CO_2_ loss and [C_2_F_5_]^−^ for most PFCAs) and has been shown to have a less than 5% false positive rate.^7,11,24,27^

### 2.2 Sample Extraction and Preparation

Microporous polyethylene tubes (mPEs) were filled with 400 mg of Sepra-ZT WAX sorbent (Phenomenex) and conditioned with methanol for 24 h, followed by conditioning with Optima-grade water (Fisher) for 48 h. The mPE configuration, prior validation, and extraction protocols can be found in Kaserzon et al. 2019.^28^ The passive sampler extracts analyzed here were part of a PFAS-monitoring wastewater study (Smolinski et al., in prep.) Samplers were suspended by stainless steel wires in the influent (*n* = 2) and effluent (*n* = 2) reservoirs of an onsite wastewater system in New York State and were retrieved after 2 weeks of deployment. After retrieval, the samplers were gently rinsed with Optima-grade water to remove particulate matter from the surface and placed into clean 15 mL polypropylene centrifuge tubes. A mixture of 21 mass-labeled PFASs (4 ng each, Table S1) was spiked onto the surface of each sampler and allowed 30 min to equilibrate. PFASs were serially extracted by sonication in 4 mL of methanol three times. Pooled extracts were combined in a new polypropylene vessel, concentrated to near dryness under nitrogen, and reconstituted in 50:50 water:methanol with 4 ng of injection standard (M4PFOA) prior to analysis. All analytical standards in the mass-labeled mix and QC standard mixture were purchased from Wellington Laboratories.

### 2.3 Targeted Data Acquisition and Processing

Samples were analyzed on an Agilent 1290 Infinity II liquid chromatography (LC) system coupled to an Agilent 6545 quadrupole time-of-flight mass spectrometer (QTOF-MS) retrofitted for PFAS-clean analysis, including the use of an Agilent PFC delay column after the solvent mixer. A binary gradient of 10 mM ammonium acetate in Optima LCMS-grade water (A) and 100% Optima LCMS-grade methanol (B) was used for chromatographic separation (Table S2). The QTOF-MS was operated in negative electrospray ionization mode (ESI−), and data were collected in DDA mode. QTOF-MS acquisition parameters are provided in Table S3.

Samples (*n* = 4) were analyzed along with three field blanks and two laboratory blanks. A quality control (QC) standard mixture (500 pg on-column; Table 1) and an analytical blank were injected before and after all samples to check for consistent sensitivity and retention times, as well as to monitor for instrumental contamination.

**Table 1:**
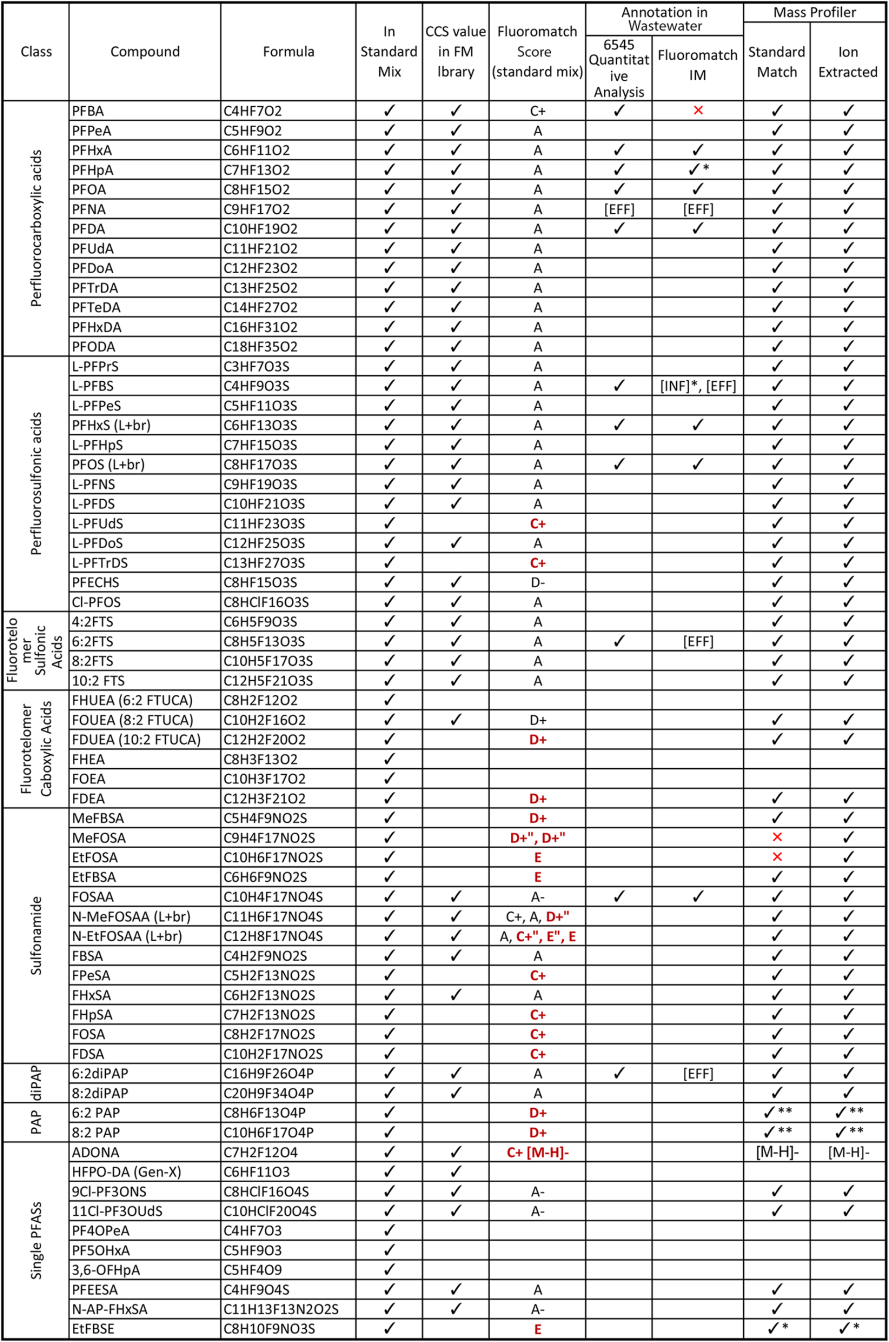
PFAS standard mix in spiked solvent and identification confidence in the standard mix and samples^a^

a Checkmarks denote the known presence in the standard mix and in the default CCS library. FluoroMatch scores are assigned automatically by FluoroMatch, where bold, red scores indicate the score assigned to features without formula annotations. “Annotation in wastewater” columns denote the presence and absence of target PFASs in wastewater samples as determined by Quantitative Analysis (Agilent 6545 data) and FluoroMatch IM workflows. Structures and full names are listed in Table S4Table S5.

* Denotes a feature annotated in one duplicate injection.

** Denotes PFAS with *m*/*z* match, but CCS values do not match literature, and ‘’denotes a feature incorrectly annotated. [INF] and [EFF] represent compounds annotated in influent or effluent only, respectively.

We previously completed targeted screening using Agilent MassHunter Quantitative Analysis software (Smolinski et al., in prep.) on the passive sampler extracts as follows. An extracted ion chromatogram list of 63 native and 19 mass-labeled (Table S1) was used as the target library. Identifications required a retention time tolerance of ± 0.20 min relative to analytical standards analyzed in the same chromatographic run and a mass tolerance of ± 10.0 ppm. PFASs detected in the extracts are listed in Table 1 (6545 Quantitative Analysis).

### 2.4 Nontargeted Data Acquisition, Processing, and Mass Profiler Analysis

Extracts were also analyzed on an Agilent 1290 LC system coupled to an Agilent 6560 ion mobility-quadrupole time-of-flight mass spectrometer (IM-QTOF-MS). Chromatography was unchanged from the targeted acquisition (Table S2). Reference mass (Agilent Technologies, Inc.) was introduced continuously via an isocratic pump to the reference nebulizer to perform continuous mass correction. Data were acquired with 4-bit multiplexing without fragmentation. A CCS calibration file was acquired with identical MS parameters at the beginning of the analytical run using ESI tuning mix (Agilent Technologies, Inc.). Solvent double blanks (*n* = 5), QC checks containing the 63 targeted PFASs (*n* = 5), and analytical blanks (*n* = 2) were analyzed before and after all sample injections. Samples included influent (*n* = 2), effluent (*n* = 2), field blanks (*n* = 3), and laboratory blanks (*n* = 2), and were each injected once.

Data were demultiplexed using the PNNL Preprocessor (v 4.1). A single-field CCS calibration was performed using MassHunter IM-MS Browser (v 10.0), and a reference mass correction was applied using MassHunter IM-MS Reprocessor (v 10.00). Features were extracted using Agilent Mass Profiler software (v 10.0.2). The minimum ion count was set to 100, and alignment tolerances were set to 0.10 min in retention time, 1.5% drift time, and 10.0 ppm. Note that these thresholds may not apply to all of the data and workflows.

Feature extraction in Mass Profiler resulted in a table of 7337 features, which was used in both the FluoroMatch IM workflow and Mass Profiler formula screening to ensure there were no discrepancies in the initial feature extraction step. Formula matching was first performed using Mass Profiler and Agilent ID Browser with the same analyte list used for targeted quantitation, including all PFASs in the standard mix (Tables 1 and S1). Formula matching parameters were defined as follows: retention time window ± 0.1 min, mass error ± 5.0 ppm, and possible negative ions including [M–H]^−^ and [M–H–CO_2_]^−^.

For the non-targeted analysis workflow (FluoroMatch IM), inputs included the aforementioned feature table, the CCS library provided with FluoroMatch IM, and the provided repeating units file. The only repeating unit set to TRUE was [CF_2_]. The FluoroMatch IM parameters were set as follows: CCS window of 2%, mass window of 0.01 Da, mass defect flag −0.11 to 0.12 (covering 90% of the EPA PFAS structure list),^14^ and polarity set to “Neg”. Results were examined in the output file and visualized in the Microsoft Power BI Desktop application provided with FluoroMatch IM. Features annotated by FluoroMatch IM were evaluated to determine false positive (FP) and false negative (FN) rates (Section 3.1) of known compounds in both the standards and wastewater samples. Features with masses and retention times of known PFASs that were not annotated by FluoroMatch IM were manually identified and included in the FP/FN rates described in the results. Handling of multiple matches due to existing isomers or in-source fragmentation (e.g., CO_2_ loss) is described in Sections S3 and S4.

Features that were not identified as target PFASs were then evaluated as follows. Average peak areas for features measured in influent or effluent were required to be ≥5× the field blank average, eliminating 2015 features. Detections with peak areas of the same order of magnitude in all sample types (analytical blanks, field blanks, standard mix, and samples) were considered to be either (i) background ions or (ii) in-source fragments of mass-labeled standards and were omitted, and features detected only in one sample were removed. Features that did not follow the expected mass-to-retention time or mass-to-drift time trends were flagged as either “RT not ordered” or “DT not ordered” and were eliminated, followed by removing features assigned an “E” score (not likely PFAS, Section 2.5,) resulting in 300 features binned into 81 homologous series. Lastly, a CCS vs. *m*/*z* filter was applied such that only features falling into the expected region for PFASs remained.^18^ In our case, the trendline CCS = 0.1799 × (*m*/*z*) + 96.308 was determined as the maximum CCS threshold as it encompassed all experimental values of confirmed, annotated PFASs. The trendline of the confirmed, annotated PFASs was CCS = 0.799 × (*m*/*z*) + 80.208. After these filters were applied, the remaining features (*n* = 16) were cross-referenced with the NORMAN list S89 Prioritization Risk database, a list of 4777 PFASs, to identify possible matches against an extensive PFAS database. This list was selected as it encompassed all available PFAS lists in the NORMAN database.^29,30^ The NTA study reporting tool (SRT)^31^ is used in the Supporting Information to evaluate the nontarget methods and results of this study.

### 2.5 Assigning Confidence Levels to PFAS Identifications Using IM-QTOF-MS

Criteria for assigning confidence were adapted from previous work.^11,32^ CCS matches within 2% of expected values were treated similarly to MS/MS spectral matches, as they provide information that helps to increase confidence in identifying a specific structure with a given *m*/*z*. As with MS/MS spectra, the usefulness of CCS for compound identification is limited by the size of the available library. However, *in silico* approaches to predict both MS/MS fragmentation and CCS are becoming more common and are considered here as potential lines of evidence to increase identification confidence.

The following confidence criteria were developed to be used postblank filtering:

**Main categories of annotation confidence and interpretation** (*Italics* means these lines of evidence require deconvoluted All Ions data, only supported using the PNNL preprocessor on Agilent files alongside FluoroMatch IM. Note that Charbonnet scales, which have been proposed for PFAS scoring as well, are included alongside FluoroMatch scores. Level 1 does not have corresponding scores in FluoroMatch (Level 1 is from targeted analysis only)):

**A**. **Confident ID** (Level 2b PFAS Confidence Level (PCL)):^33^ Proposed structure based on accurate mass match^1^ and CCS and/or *class-specific rule-based annotation using fragmentation pattern*(3)*and/or confirmation using standards*.

**B**. **Tentative ID** (Level 2c PCL): *A structure may be proposed based on the exact mass and the presence of fragments common to PFASs.*(4)*These compounds are likely to be PFASs because common fluorine-containing fragments are observed, but additional information or manual review is necessary to confirm the structure.*

**C**: **Possible ID** (Level 2c PCL if the homologous series includes another member that is scored A; otherwise, Level 3d, with visual confirmation of consistent retention time and CCS or drift time vs *m*/*z* trend using FluoroMatch Visualizer): Proposed PFAS subclass based on the identification of other PFASs (Level A or B) within the same homologous series.^5^

**D**: **No ID** (Level 5 PCL): Possible PFAS based on the presence in a homologous series, exact mass match, formula prediction, or mass defect filtering.^6^ These features should also fall within an *m*/*z* vs. CCS region that is consistent with PFASs. These compounds might be PFASs, but structural information is not known. More information is needed for identification.

**E**: **Likely not a PFAS** (Level 5 PCL): No evidence of PFAS-associated MS/MS fragments, exact mass matches, homologous series, CCS matching, or mass defect.

#### 2.5.1 Definitions

*Exact mass match*: Match to the exact *m*/*z* ratio (within a specified parts-per-million threshold) of a formula in the EPA PFAS Master List or from compounds used as standards or found in literature.
*CCS match*: A match within at least 2% of a database value based on analyzed standards of known composition (not predicted CCS values)
*Subclass-specific fragmentation pattern*: In the case of FluoroMatch, this refers to the detection of the most abundant fragment corresponding to a given subclass, regardless of relative intensity. These subclass-specific fragments are based on annotated MS/MS data from standards or literature for the given PFAS subclass.
*Fragments common to PFAS*: In the case of FluoroMatch, this refers to fragments that have been associated with substructures of PFASs based on standards and literature. For example, all PFASs containing a C_3_F_6_ chain or longer would have [C_3_F_5_]^−^ as a possible common fragment. These substructure-based fragmentation rules were applied to the entire EPA master list to predict fragments for all species.
*Homologous series*: Based on Kendrick mass defect (KMD) plots with masses normalized to CF_2_, these are series of two or more molecules separated by the mass of one or more CF_2_ units and falling under the same CF_2_ normalized mass defect. Homologous series must also follow the correct retention time order (molecules included in the same subclass with higher molecular weights should elute later, assuming reverse-phase chromatography).
*Mass Defect Filtering*: A mass defect range of −0.11 to 0.12 is used, which was quantitatively determined to cover the mass defect of 90% of the PFASs in the EPA PFAS structure list.^11^ Note that certain PFASs, such as those containing mercury, iodine, other elements, or minimal fluorination, may not meet this criterion but may still be annotated using the further evidence described above.

#### 2.5.2 Subcategories of Annotation Confidence and Interpretation

**A+** = Confident ID, using standards (not applicable to FluoroMatch software; applicable to targeted methods)

**A** = Confident ID (CCS or class-based fragmentation observed along with exact mass) and 2+ in homologous series

**A-** = Confident ID (CCS or class-based fragmentation and exact mass)

***B+****= Tentative ID, highly likely PFAS (1+ PFAS fragments from standards and exact mass) and 2+ in a homologous series*

***B****= Tentative ID, likely PFAS (1+ PFAS common fragment (F-containing) and exact mass) and 2+ in a homologous series*

***B-****= Tentative ID, likely PFAS (1+ PFAS fragments from standards and exact mass OR 1+ common PFAS fragment (F-containing) and exact mass)*

**C+** = Possible ID, possible PFAS: 2+ in homologous series, and at least one confident PFAS identification within the homologous series (A grade or higher)

***C****= Possible ID, possible PFAS: 3+ in homologous series, and at least one highly likely PFAS identified within the homologous series (B grade or higher)*

***C-****= Possible ID, possible PFAS: homologous series 2+ within, and at least one highly likely PFAS identified within the homologous series (B grade or higher)*

**D+** = No ID; possible PFAS: mass defect falling within −0.11 and 0.12 OR exact mass match, and 3+ within a homologous series

**D** = No ID, possible PFAS: 3+ within homologous series

**D-** = No ID, possible PFAS: Mass defect falling within −0.11 and 0.12, OR exact mass match

**E** is likely not PFAS

## 3 Results and Discussion

### 3.1 Validation and False Positive/False Negative Rates for FluoroMatch IM

Note that we determined our false positive and false negative rates using data acquired on an Agilent 6560 DTIMS system. FluoroMatch IM is vendor-neutral, and user CCS libraries can readily be added or swapped with the default CCS libraries depending on the vendor, if required by users. The built-in libraries were developed using CCS values acquired on 6560 systems and were found to show good agreement with CCS values obtained from traveling wave and drift tube ion mobility systems across various platforms (Agilent 6560,^34^ Waters Synapt T-Wave,^17^ Waters VION T-Wave,^35^ and Waters Cyclic Ion Mobility).^36^ Of the 54 compared values from four publications, 50 (93%) were within 2% of the CCS values in FluoroMatch IM’s built-in libraries. Note that three other studies using the instruments mentioned above did not show good agreement,^37−39^ suggesting the importance of accurate CCS calibration and validation, especially in traveling wave systems, as has been discussed previously.^17,21^ All data for the CCS comparison are shown in Table S5 and Figure S1.

To evaluate the false positive and false negative rates using FluoroMatch IM, 63 PFAS standards (Table 1) were spiked into methanol and water (1:1 v/v), and data were analyzed by the FluoroMatch IM workflow. Of the 63 spiked PFASs, 42 had CCS entries included in the FluoroMatch IM library. Only these 42 PFASs were evaluated to determine false positive rates, as CCS matching could only be performed for these compounds. Table S6 includes all results from FluoroMatch IM. Overall, 66 of the 7337 features detected in standards were annotated, meaning that they were assigned a formula and compound name by FluoroMatch IM. For an annotation at any confidence level to be made, an *m*/*z* match is required. Forty-seven features received an A or A– score, indicating that there was both a CCS and exact mass match. Seven of these were [M–H]^−^ adducts of already identified PFCAs by the [M–H–CO_2_]^−^ adduct, and three PFASs (FBSA, PFHxA, and PFNA) eluted at two retention times, resulting in duplicate features. This resulted in a total of 37/42 PFASs confidently (A or A– score) annotated by FluoroMatch IM.

Sixteen of the annotated features received a C+ score, 11 of which fell into the PFCA homologous series. For ten of these annotations, the CCS value matched the [M–H]^−^ adduct, but the *m*/*z* matched the [M–H–CO_2_]^−^ adduct, suggesting that a CO_2_ neutral loss occurred post drift time measurement, resulting in a CCS mismatch by the software. The 11th additional PFCA was PFBA, which only received a C+ score despite an exact *m*/*z* and CCS match. Three of the C+ scores were assigned to isomers of PFOS and PFHxS, and the remaining two were assigned to isomers of N-EtFOSAA and N-MeFOSAA. Of the six remaining annotations, 3 were assigned incorrectly to FOSAA with D+ scores, 1 was assigned incorrectly to EVE Acid with a D+ score, and two were correctly assigned (8:2 FTUCA, D+; PFECHS, D−). Across all 66 annotated features (42 unique formulas), 4 incorrect assignments (2 unique formulas) were made, each of which was assigned a D+ score warranting manual confirmation, for a false positive rate of 6% by feature count or 5% by formula count. The false positive rate for features assigned high-confidence scores of A, A–, and C+ (C+ scores are highly confident after assessing RT and DT trends in the Visualizer for outliers) was 0%*.*

Of the 42 CCS entries available, 40 were correctly assigned (Table 1). The remaining two PFASs that were not assigned were ADONA [M–H–CO_2_]^−^ and Gen-X, neither of which were extracted by Mass Profiler upstream. Of the features that were extracted by Mass Profiler and had an available CCS library entry (*n* = 40), the false negative rate was 0% for the FluoroMatch IM workflow. Three PFASs were correctly identified but with low scores (PFBA, 8:2 FTUCA, and PFECHS). 8:2 FTUCA was correctly annotated by accurate mass and homologous series but was assigned a D+ score due to a CCS mismatch between the library value (192.4 Å^2^) and the measured value (172.2 Å^2^). PFECHS received a D+ score, and PFBA received a C+ score. In both cases, the mass error and CCS error were within set thresholds. These lower scores were due to library entry issues or algorithm issues that will be resolved in the next software release.

**Figure 4:**
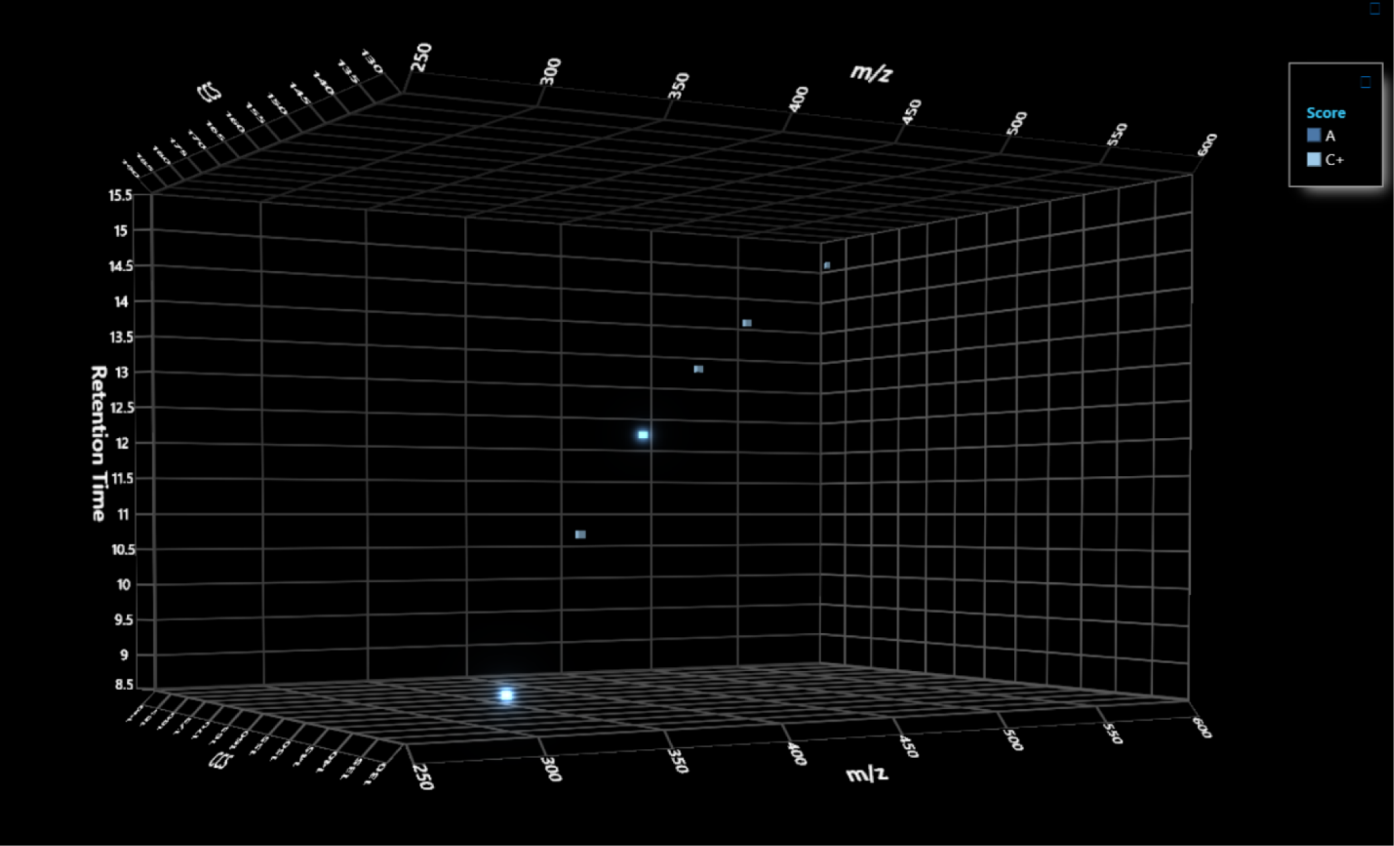
Perfluoroalkyl sulfonamide (FASA) series. FBSA and FHxSA highlighted to denote ″A″ identifications for which CCS measurements were available.

Every feature in the feature table is assigned a score, regardless of whether the feature is a suspected PFAS or not (Section 2.5). The 21 remaining PFASs in the standard mix that were not included in the default CCS library were assigned as follows. Ions for 6 of the 21 remaining PFASs were not extracted via Mass Profiler and thus could not have been identified via FluoroMatch. These ions were likely not detected in the raw data files due to poor amenability to the LC-MS method. Six PFASs were assigned C+ scores, indicating that they fell within a homologous series with at least one high-confidence CCS match. While no CCS values were available in the library for these six PFASs, they were correctly assigned after manual review of chromatographic and drift time trends using the Visualizer. The six PFAS exhibited consistent retention time and CCS order with other members of their respective homologous series and could be confidently identified as members of PFSA (*n* = 2, Figure 3) and perfluoroalkyl sulfonamide (*n* = 4, Figure 4) series, highlighting that inclusion in a homologous series can increase identification confidence even without library entries.^32^ Furthermore, leveraging trendlines of *m*/*z* vs RT order and CCS order increased confidence in PFAS identifications, especially when additional identifiers like fragmentation are not available for a given feature.^18,19,32,40^

Six extracted ions received D+ scores, and three received E scores. EtFBSA, EtFOSA, and EtFBSE were assigned E scores without annotation, as they were not part of a 3+ homologous series and their formulas were not included in the CCS library. MeFOSA was incorrectly identified as the [M–H–CO_2_]– adduct of FOSAA with a D+ score, which denotes an exact mass match. However, the D+ score warranted further validation, at which point it was determined that neither the retention time nor the CCS value was consistent with FOSAA, and the identification was corrected.

Overall, a false negative rate of 27% was obtained for the entire workflow (feature extraction + FluoroMatch IM), regardless of a CCS library entry and subsequent assigned score; 17 of 63 standards were not correctly annotated or binned within a homologous series, with at least one member containing a CCS match. Two PFASs were incorrectly annotated (FOSAA, EVE acid), and three additional features were incorrectly assigned formulas despite those formulas already being confidently assigned to other features (N-MeFOSAA, N-EtFOSAA). The incorrect duplicate annotations were easily ruled out, as they were assigned low-level scores, and abundances in standard injections were low or zero. It is important to note that these false negative and false positive rates may change depending on the analytes being investigated, sample matrix, analyte concentration, and workflow thresholds. If only considering compounds with extracted features (hence removing false negatives not related to FluoroMatch IM), the false negative rate was 16% (9/55), and the false positive rate was 2% (1/55). The CCS library has been expanded to include all molecules analyzed in this study and will continue to be expanded by users over time, enabling additional annotations with higher confidence in the future.

In summary, of the 63 targeted PFASs (spanning 11 subclasses), 7 PFASs were not extracted by feature extraction upstream of FluoroMatch IM. Of the remaining 56 PFASs, 40 were correctly annotated with A/A– (*n* = 37), C+ (*n* = 1), D– (*n* = 1), or D+ (*n* = 1) scores. Of the unannotated PFASs, six were assigned C+ scores based on homologous series, six (11%) were assigned D+ scores, and three (5%) were assigned E scores. One PFAS was incorrectly annotated with a D+ score (MeFOSA) but was manually corrected as recommended by the scoring scheme.

### 3.2 Comparison of Targeted Screening and FluoroMatch IM Workflows

Using Mass Profiler and traditional targeted analysis, 54 of 63 target PFASs were annotated in the standard mix, and 11 PFASs were identified in wastewater samples (Table 1, Mass Profiler). In comparison, FluoroMatch IM correctly annotated 40 targets in the standard mix, with an additional seven manually annotated after being listed as part of a homologous series by the software (47/63). In wastewater, 11 of the 12 previously identified PFASs were correctly annotated via FluoroMatch IM (Section 2.3 and Table 1). The twelfth known PFAS in wastewater (PFBA) was not annotated via Mass Profiler or the FluoroMatch IM workflow, as it was not extracted upstream via Mass Profiler. There was one false positive annotation by FluoroMatch IM in the wastewater samples (N-MeFOSAA), likely due to in-source fragmentation of a later-eluting unknown.

Overall, all target PFASs identified in wastewater via Mass Profiler were also identified via FluoroMatch IM. In the standard mix, the Mass Profiler workflow performed better than FluoroMatch IM due to the extracted ion target list containing masses and retention time values for all targets. With the continued expansion of the FluoroMatch IM CCS library, annotation coverage is expected to continue to improve. It is also worth noting that known retention times were used as an identifier in the targeted Mass Profiler workflow, which cannot be performed for unknowns, whereas the leverage of CCS versus *m*/*z* space can be employed independently of known retention times and fragmentation information. Furthermore, users can readily expand the CCS libraries to include known targets. Compound annotations by all three workflows are shown in Table 1.

### 3.3 Discovery of Additional PFASs Using FluoroMatch IM for Wastewater Analysis

After filtering the FluoroMatch IM results (Section 2.4) there were 16 features within ten homologous series of interest beyond targeted PFASs (Table S7). Of the 16 features, 9 were assigned D+ scores, and 7 were assigned D scores. One series (series 116) was tentatively identified as 6:2, 7:2, and 8:2 fluorotelomer dihydrogen phosphates (PAPs), though experimental CCS values acquired in this study do not agree with predicted and experimental values in the literature.^18,26^ Both 6:2 and 8:2 PAP were included in the standard mix and matched via Mass Profiler; however, uncertainty lies in their annotation due to CCS mismatches. Series 118 (Tables S6 and S7, *n* = 3) *m*/*z* values matched those of fluorinated carbon chains (PFAA fragments) but did not align with retention times of targeted PFASs and therefore could not be ruled out as in-source fragments and remain unknowns of interest. While these 16 features are all level 5 identifications^40^ and are tentative, they are flagged as likely PFASs and would have been overlooked without a nontarget workflow screening for homologous series.

**Figure 5:**
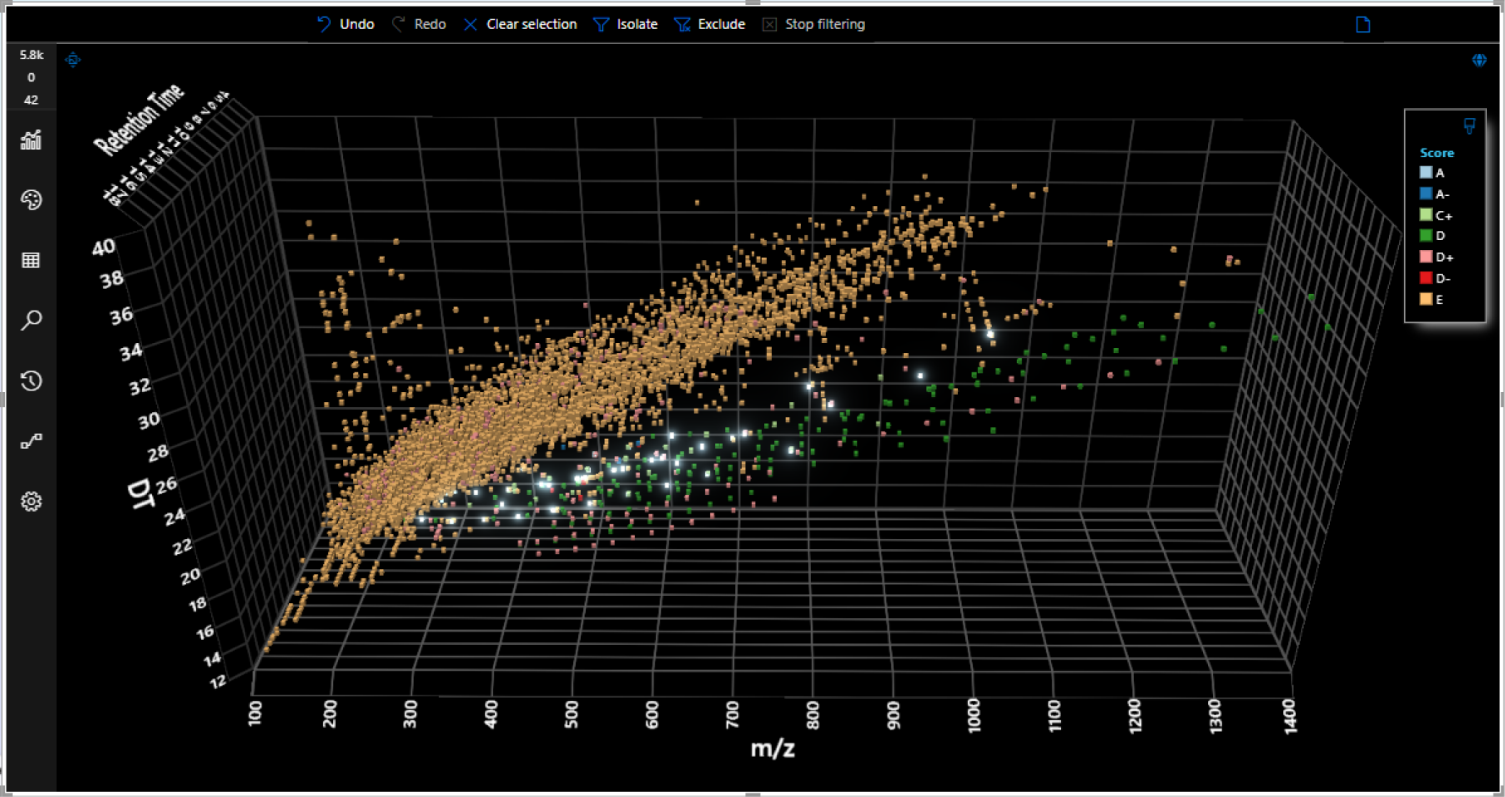
3D plot of all features extracted by Mass Profiler in the sample set, binned by the assigned score from FluoroMatch IM. Features assigned an “A” score are highlighted. A region consisting of all potential PFAS (based on FluoroMatch score between A through D) is clearly distinguished from cluster of score E features.

While structural annotation of PFASs is ideal, when database entries are not available, it is still important to decipher whether a feature is likely to be a PFAS or not. Nontargeted methods for prioritizing the likelihood of a feature being a PFAS, utilized by FluoroMatch, include mass defect flagging, homologous series assignment, position in Kaufmann space (isotopic ratios), formula prediction, and database matching based on accurate mass and/or CCS values. Kaufmann space (described previously)^41^ refers to the ratio of the mass defect over the equivalent carbon number versus the *m*/*z* over the equivalent carbon number. The equivalent carbon number is estimated using the M + 1 isotopic peak percentage. PFASs will have a high mass compared to the equivalent carbon number relative to biological and organic compounds, due to the high mass of F vs. H, and will have a negative mass defect, therefore falling in a unique region of the Kaufmann space as compared to non-PFAS species. Features assigned a score ranging from A to D are potential PFASs based on various metrics (Section 2.5), whereas features assigned E are likely not PFASs. Figure 5 below shows a 3D plot colored by FluoroMatch score across drift time, *m/z,* and retention time of features detected in standards and wastewater injections (overlaid). Many features assigned E (likely not PFASs) are clearly separated from the region of high-confidence annotations (A or A-) (Figure 5), consistent with previous literature showing the separation of halogenated organic molecules from biomolecule space.^18^ Hence, using 3D scatter plots with FluoroMatch IM Visualizer and FluoroMatch scoring, features can readily be prioritized as likely to be PFASs in complex samples. Future developments in the next release will focus on leveraging this space to automatically determine the PFAS likelihood based on trends observed in Figure 5.

Overall, FluoroMatch IM highlights the potential of CCS measurements for increasing the confidence of novel PFAS identifications in samples containing trace levels of contamination, where in-depth analysis of MS/MS spectra proves difficult. Furthermore, fragmentation data can be incorporated using the PNNL Preprocessor^24^ to deconvolute All Ions data for Agilent QTOF files. As a vendor-neutral solution with demonstrated cross-platform compatibility for LC-IM-MS/MS analysis, we expect this software to accelerate many key discoveries in PFAS research over the coming years.

#### 3.3.1 Implications

The potential of IMS as an orthogonal technique for prioritization of likely PFASs and other xenobiotics has only recently been demonstrated in environmental and biological samples.^18^ As such, CCS values were not considered among the lines of evidence included in systems previously proposed for communicating identification confidence,^2,32^ though recent works have incorporated CCS values into confidence schemes in both general^22^ and PFAS-specific applications.^40^ Incorporating ion mobility and CCS matching as a line of structural evidence increases confidence in annotations and reduces false positive rates.^22^ CCS values are undoubtedly useful for differentiating PFAS isomers that are traditionally challenging to separate^19^ (e.g., branched isomers). Traditionally, differentiation of coeluting isomers is done by examining MS/MS fragmentation; however, due to the stability of perfluorinated moieties, some PFASs do not readily generate informative fragments at typical collision energies (e.g., unsaturated PFOS versus PFEtCHxS).^32,42^ Furthermore, MS/MS fragmentation varies among instrument types and depends on collision energy, complicating the use of large fragmentation libraries and *in silico* predictions across instrument platforms.

Here, we demonstrate the utility of FluoroMatch IM’s automatic CCS-matching to a user-generated library as a promising line of evidence in addition to (or in place of) MS/MS fragmentation matches. This was demonstrated by a false positive rate of 5% when CCS and *m*/*z* matching were required, even in the absence of MS/MS and retention time inputs. Given that CCS values are not highly variable among instruments, as described here and in the literature,^17,21^ the defined CCS vs. *m*/*z* area of halogenated organics is expected to be fairly consistent among different IMS instrumentation, unlike retention time vs. *m*/*z* trends, which change depending on, e.g., chromatographic gradient. Further, distinct CCS vs. *m*/*z* trendlines of PFAS subclasses are well-characterized and rely on near-constant molecular properties.^19^ This allows rapid prioritization of potential PFASs before detailed annotation.

## Data Availability

Data is accessible on MassIVE: The raw data files can be found here: ftp://massive.ucsd.edu/v07/MSV000096605/ Simply use Finder in Windows, copy the FTP link to the address bar to access the files, and drag them onto your computer. The interactive dataset for this manuscript can be accessed online (no software required) via innovativeomics.com/datasets.

## Supporting Information Available

FluoroMatch IM with All Ions data; FluoroMatch IM with SLIM data; handling of common neutral losses in the PFAS identification workflow; CCS values of PFASs reported in literature; PFCA homologous series; isomer differentiation (PDF)
Mass-labeled PFAS included in this study; acquisition parameters of Agilent 6545 and Agilent 6560 mass spectrometers; binary gradient elution program of LC/MS analysis. A = 10 mM Ammonium acetate in methanol, B = Optima LCMS-grade water; data for the CCS comparison; PFAS formulas, acronyms, and structures; inal novel features; expansion of Table 1 to inlude specific feature numbers found in the feature table and experimental retention time values; comparison of CCS values across reported literature values (XLSX)
NTA Study Reporting Tool guidelines(PDF)
