# Supplementary material for: FluoroMatch IM: An Interactive Software for PFAS Analysis by Ion Mobility Spectrometry
Source: Environ Sci Technol. 2025 Mar 25;59(13):6636–48. doi: 10.1021/acs.est.4c13846 (PMC11984190; doi:10.1021/acs.est.4c13846)
Supplement: Supplementary file 1 — es4c13846_si_001.pdf [file es4c13846_si_001.pdf]

Supplementary information for es-2024-13846w

FluoroMatch IM: An Interactive Software for PFAS Analysis by Ion Mobility Spectrometry

Rachel Smolinski[1a], Jeremy Koelmel[2a], Paul Stelben[2], David Weil[3], David Godri[4], David Schiessel[5], Michael Kummer[5], Sarah M. Stow[3], Sheher Mohsin[3], Lauren Royer[6], Alan McKenzie-Coe[6], Thomas Lubinsky[6], Daniel DeBord[6], Olivier Chevallier[3], Emma E. Rennie[3], Krystal J. Godri Pollitt[2*], Carrie McDonough[1*]

[1] Department of Chemistry, Carnegie Mellon University, Pittsburgh, PA, 15213, USA
[2] Department of Environmental Health Science, Yale School of Public Health, New Haven, CT, 06520, USA
[3] Agilent Technologies, Inc., Santa Clara, CA, 95051, USA
[4] 3rd Floor Solutions, Toronto, ON, CA 43964
[5] Innovative Omics, Inc., Sarasota, FL, 34235, USA
[6] MOBILion Systems, Inc. Chadds Ford, PA, 19317, USA

[a] Shared first authorship
*Shared corresponding authorship

Contact:

Krystal J. Godri Pollitt: krystal.pollitt@yale.edu

Carrie McDonough: cmcdonou@andrew.cmu.edu

Summary: 5 pages, 2 figures, 8 tables

Table of Contents:

S1: FluoroMatch IM with all ions data S3

S2: FluoroMatch IM with SLIM data: S3

S3: Handling of Common Neutral Losses in the PFAS Identification Workflow S3

Figure S1: CCS values of PFASs reported in literature S4

Figure S2: PFCA homologous series S4

S4: Isomer Differentiation S5

S1: FluoroMatch IM with all ions data:
FluoroMatch Modular includes *in silico* MS/MS fragment libraries (fragment annotations and masses, not intensity) for over 200,000 PFAS chemical species including biotransformation products, rule-based MS/MS fragment libraries developed using chemical standards with a less than 5% false positive rate covering over 10,000 chemical species, and fragment screening, alongside all other features described in-depth previously.[6,10,20]

S2: FluoroMatch IM with SLIM data:
Additional testing using a MOBILion Systems MOBIE[(R)] high resolution ion mobility device was performed to evaluate cross-platform compatibility of FluoroMatch IM. Please see **https://www.rafa2024.eu/pdf/Program%20RAFA%202024_final%20B5%20web.pdf**

S3: Handling of Common Neutral Losses in the PFAS Identification Workflow:

For most PFCAs, an $[M\text{-}H]^-$ and an $[M\text{-}H\text{-}CO_2]^-$ entry is present in the CCS library, and Fluoromatch IM reports the $[M\text{-}H]^-$ and $[M\text{-}H\text{-}CO_2]^-$ adducts as two differentiable homologous series. The $[M\text{-}H\text{-}CO_2]^-$ adduct was identified with an A score for all target PFCAs except for PFBA (C4) and PFODA (C18), and the $[M\text{-}H]^-$ adduct was identified with an A score for C9 – C14, C16, and C18 PFCAs. Note that in section 3.3, only one adduct was considered in the validation (each PFAS only counted once). However, an additional 10 $[M\text{-}H\text{-}CO_2]^-$ features were matched with C+ scores, each for a targeted PFCA that was already identified with an A score, and with all $[M\text{-}H\text{-}CO_2]^-$ adducts falling in the same homologous series. The strategy for differentiation between the duplicate $[M\text{-}H\text{-}CO_2]^-$ identifications across the PFCA series is use of the CCS value, except for PFHpA. For PFHpA, one of the duplicate $[M\text{-}H\text{-}CO_2]^-$ identifications was found at a lower retention time (-2.2 minutes), indicative of an in-source fragment of another unknown PFAS to form PFHpA. This could easily be corrected based on retention time trends for the PFCA sub-class. For the remaining PFCA duplicates (C9-C14, C16, and C18), there are two $[M\text{-}H\text{-}CO_2]^-$ adducts that have the same m/z and retention time, but differing CCS values. Feature details are listed in Table S2. Figure 5 depicts the $[M\text{-}H\text{-}CO_2]^-$ series of all identified PFCAs, where there is a clear differentiation between the measured drift times across the series. The multiple observed drift times per chromatographic feature in this series is likely indicative of CO_2 losses occurring at different points in ion transmission, likely (i) in-source (before drift tube), and (ii) post IM separation. This is also supported by the comparison of CCS values of the $[M\text{-}H]^-$ adduct agreeing with the CCS values of the second $[M\text{-}H\text{-}CO_2]^-$ adduct, as shown in Figure 5.

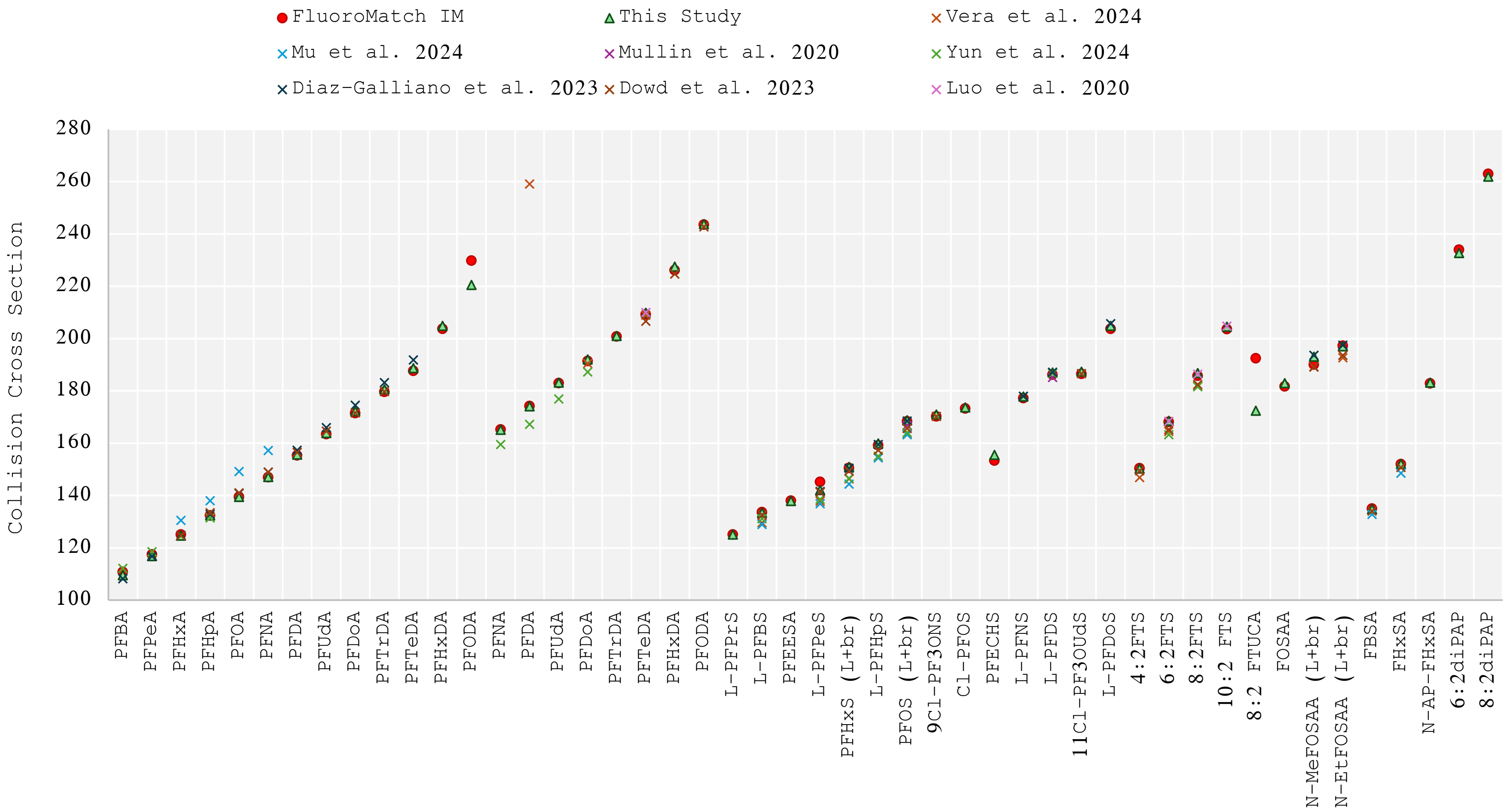

Figure S 1: CCS values of PFASs reported in literature

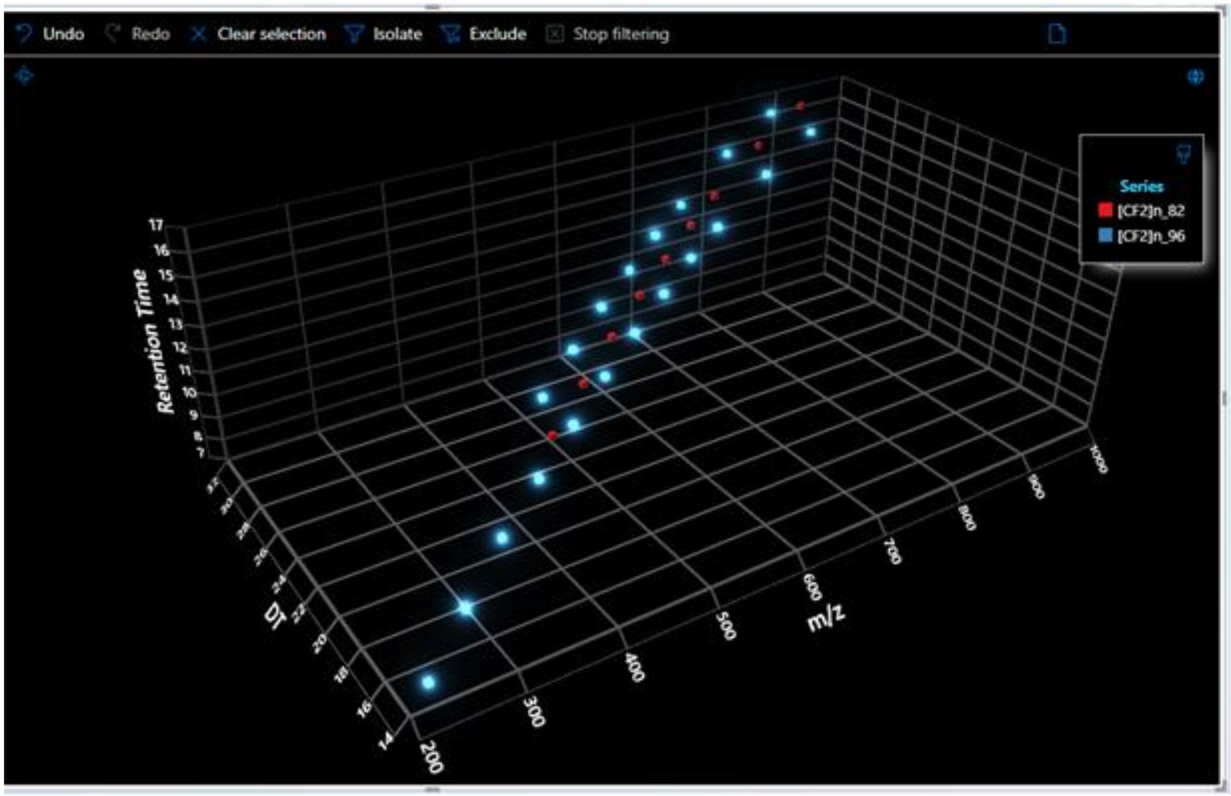

Figure S 2: PFCA homologous series. Red series (n_82) represents $[M\text{-}H]^-$ PFCA adducts and the blue, highlighted series represents the $[M\text{-}H\text{-}CO_2]^-$ adducts. CCS, drift time, m/z, retention time, and formulas are listed in Table S2.

S4: Isomer Differentiation

One PFHxS, two PFOS, and one N-MeFOSAA branched isomers were identified as separate features from their linear counterparts with C+ scores. These isomers are assigned C+ due to the lack of CCS values in the library; future inclusion of CCS values for branched isomers into the FluoroMatch IM PFAS libraries could be used to readily identify these isomers. This would be highly advantageous given that fragmentation evidence can rarely be used to identify these isomers, and their accurate masses are identical. Even if isomers are not contained in the CCS libraries, the automatic detection of these isomers via homologous series detection makes it relatively facile to annotate them after examining retention time and drift time trends (branch isomers generally appear earlier in both dimensions, when using reverse phase chromatography).
