# Supplementary material for: FluoroMatch IM: An Interactive Software for PFAS Analysis by Ion Mobility Spectrometry
Source: Environ Sci Technol. 2025 Mar 25;59(13):6636–48. doi: 10.1021/acs.est.4c13846 (PMC11984190; doi:10.1021/acs.est.4c13846)
Supplement: Supplementary file 3 — es4c13846_si_003.pdf [file es4c13846_si_003.pdf]

Thank you for using the SRT!
Please use the information below to cite or acknowledge the SRT.

We (BP4NTA members) hope that it promotes improved reporting practices for NTA research. To enable future assessment of its impact on NTA reporting, we encourage manuscript authors and reviewers to follow the citation/acknowledgement instructions below. To enable future updates to the SRT, please visit www.nontargetedanalysis.ort/SRT to provide feedback.

MANUSCRIPT AUTHORS:

If the SRT was used during manuscript preparation, please describe its use in the Methods section of your manuscript and cite both the Peter et al. manuscript and the SRT itself (PDF or Excel).

Template Language: The NTA Study Reporting Tool (SRT) was used in the preparation of this manuscript (Peter et al., 2021; 10.6084/m9.figshare.19763482 [PDF] or 10.6084/m9.figshare.19763503 [Excel]).

MANUSCRIPT REVIEWERS & EDITORS:

If the SRT was used during manuscript review, please encourage manuscript authors to acknowledge its use by including the DOIs in the Acknowledgements section of their manuscript. Optional template language to use in reviewer or editor comments is provided below. The filled SRT is an anonymized file and can be attached to reviewer comments and provided to authors directly.

Template Language: The NTA Study Reporting Tool (SRT) was used in the review of this manuscript and is attached to reviewer comments. More information on the SRT can be found at www.nontargetedanalysis.org/SRT. To enable future assessment of its impact on NTA reporting, please acknowledge its use in the Acknowledgements section of your manuscript: "*The NTA Study Reporting Tool (SRT) was used during peer review to document and improve the reporting and transparency of this study (10.1021/acs.analchem.1c02621; 10.6084/m9.figshare.19763482 [PDF] or 10.6084/m9.figshare.19763503 [Excel]).*"

RELEVANT CITATIONS:

Peter, Katherine T., et al. "Nontargeted Analysis Study Reporting Tool: A Framework to Improve Research Transparency and Reproducibility." *Analytical Chemistry* 93.41 (2021): 13870-13879. DOI: 10.1021/acs.analchem.1c02621

BP4NTA (2022): NTA Study Reporting Tool (PDF). figshare. DOI: 10.6084/m9.figshare.19763482

BP4NTA (2022): NTA Study Reporting Tool (Excel). figshare. DOI: 10.6084/m9.figshare.19763503

NTA Study Reporting Tool

Please read before using!

Purpose: This Tool was developed for use by NTA researchers and reviewers to assess the quality of NTA study reporting. The resulting scores reflect solely whether the reporting is sufficiently complete and transparent (based on current, best available understanding of key study aspects in the environmental, food, exposomics, and metabolomics NTA communities). The Tool is not intended for evaluating the quality of the study or resulting data. The primary goal of using the SRT is to ensure that researchers provide all information about the study in sufficient detail to understand method details, replicate the analyses, enable comparisons across studies, and evaluate how study design choices may impact results.

We also encourage two supplementary uses of the Tool: 1) to guide study design - by considering what should be reported, a researcher is inherently encouraged to incorporate the necessary aspects into their study design, and 2) as a portal to relevant reference content and resources, which are available at the BP4NTA website (www.nontargetedanalysis.org) and via active hyperlinks within the SRT.

Notes & Guidance: The "Example Information to Report" column provides a brief list of representative items relevant to each sub-category - not all are required or necessary for every study, especially given differences across studies in different fields (e.g., environmental vs. metabolomics) and varied study goals (e.g., toxicant discovery vs. biological analysis). Researchers and reviewers should use their expertise and discretion to determine which aspects pertain to a given study, and whether additional details beyond those explicitly listed are also critical to report. Additionally, certain sub-categories may not be relevant to a given study (hence the option to select "NA"), or may be less critical to the overall quality and completeness of reporting. To evaluate these aspects, we strongly encourage users to consider the study type and objectives (e.g., method development, performance evaluation, field application), as well as conceptual linkages across subcategories (e.g., between Statistical Analysis and Statistical Outputs). We also encourage reviewers to include a rationale, so that authors/researchers may readily address concerns. Please also note that the Sections (Methods and Results) are not intended to indicate the location in a manuscript where the information is reported - a user should consider the manuscript in its entirety (including any supporting documents and/or citations).

Scoring: *NA* = not applicable (gray); *3* (blue) is the highest score and *0* (red) is the lowest. See score explanation table for details (www.nontargetedanalysis.org/srt/#srt-score-table and summarized below).

Toggle to show score colors vs. fillable fields

Section	Category	Sub-Category	Example Information to Report	Score (drop-down menu) NA 0 1 2 3	Rationale for score
Methods	Study Design	Objectives & Scope	• Study goals and hypotheses<br>• Scope of the study with respect to use of NTA / suspect screening<br>• Expected chemical and/or metabolite coverage of the approach and potential limitations	2	Scope of study with respect to NTA is defined, potential limitations are described throughout methods and results.
		Sample Information & Preparation	• Sample collection/replication, handling/storage, preparation, extraction, & clean-up methods (and related QA practices)<br>• Intended use of samples (e.g., method development, compound identification, etc.)<br>• Development and intended use of blanks	3	Sample collection and preparation followed established protocols and representative blanks were analyzed with real samples.
		QC Spikes & Samples	• Development of QC spikes/samples (e.g., isotopically labeled standards/spikes, native standard spikes, matrix pools)<br>• Intended use of QC spikes/samples (e.g., to monitor instrument performance, data normalization, evaluate signal response range, etc.) and associated calculations and/or visualizations (e.g., mass error of spiked QC compounds, plot of signal response over time, etc.)	2	QC spiked were analyzed to monitor instrument performance, though they were not matrix-matched or pooled.
	Data Acquisition	Analytical Sequence	• Sample randomization and use of replicate injections (e.g., technical/analytical replicates, field/laboratory/biological replicates)<br>• Inclusion of blanks and QC samples in the acquisition sequence<br>• Information about single vs. multiple analytical batches	3	Samples were analyzed in a single batch, were randomized in order, and were analyzed with CQ spikes, analytical blanks, and field blanks.
		Chromatography	• Note: chromatography is intended to include any online separation technique used prior to mass spectrometric detection.<br>• Instrument specifications<br>• Method settings (e.g., column/guard, mobile phases, gradient, injection techniques)	3	Instrument and method parameters were well described.
		Mass Spectrometry	• Instrument specifications<br>• Instrument calibration and/or tuning procedures<br>• Method settings (e.g., acquisition parameters, such as polarity, resolution, data-dependent vs. data-independent)	3	Instrument and method parameters were well described.
	Data Processing & Analysis	Data Processing	• File conversion information (e.g., to open-source format, centroiding)<br>• Software program(s) used<br>• Workflow steps (e.g., peak picking, RT calibration, alignment, gap filling) and settings<br>• Feature detection thresholds (e.g., replicate detection criteria; min height, area, or S/N levels; comparison to occurrence/abundance in blanks)<br>• Data correction or normalization methods (e.g., peak area/height normalization or scaling, blank subtraction)	3	Data processing and feature prioritization are described throughout the text.
		Statistical & Chemometric Analysis	• Software programs(s)/package(s) used & samples/sample groups to which analyses were applied<br>• Basic statistical analysis method goals (e.g., summarize data, evaluate variability, hypothesis testing, identify outliers), type (e.g., Wilcoxon rank sum test, Chi-square test, dispersion ratio evaluation, Spearman/Pearson evaluation), assumptions, and settings/thresholds<br>• Chemometric analysis method goals (e.g., prioritize features, compare/classify samples, evaluate relationships between features), type (e.g., differential analysis, principal component analysis, hierarchical clustering, dimensionality reduction, metabolomic pathway analysis), assumptions, and settings/thresholds	NA	Purpose of the study was primarily in workflow validation and identification of PFASs in wastewater. Comparisons were not made between sample groups.
		Annotation & Identification	• Software program(s) used (or description of manual annotation/identification efforts)<br>• Libraries and databases used (including details such as chemical coverage, resolution, metadata inclusion; information about in-house databases)<br>• Workflow steps (e.g., formula assignment, suspect screening, MS/MS spectral interpretation or library matching)<br>• Workflow methods & settings (e.g., formula prediction method, scoring algorithms; mass error/RT tolerances, accepted match scores)	3	Workflow steps and settings are well-described.
Results	Data Outputs	Statistical & Chemometric Outputs	• Basic statistical outputs (e.g., adj. p-values, standard deviations, test statistics)<br>• Chemometric analysis results (e.g., reported classifications/groupings of features/samples, outlier removal, observed data trends, metabolomic pathway analysis)<br>• Visuals/plots (e.g., Venn diagrams, heatmaps, clustering dendrograms, volcano plots, box plots, network diagrams, PCA & loading plots, molecular network diagrams)<br>• New statistical metrics, algorithms, packages, and/or scripts	NA	Purpose of the study was primarily in workflow validation and identification of PFASs in wastewater. Comparisons were not made between sample groups.
		Identification & Confidence Levels	• Reported identifications and associated confidence levels (e.g., levels described by Schymanski et al., *ES&T*, 2014)<br>• Supporting data for annotation/identification (e.g., formula match scores, fine isotope pattern, retention time match, MS/MS match scores, source of MS/MS spectra)<br>• For features with lower confidence IDs (i.e., not standard-confirmed), proposed tentative structures and other annotated data<br>• Semi-quantification or quantification data<br>• Exported MS/MS spectra (e.g., as a library, database, or deposition into online repository)	3	Semi-quantification of data was not considered in this study, though remaining elements were well described and confidence of identifications is clear throughout.
	QA/QC Metrics	Data Acquisition QA/QC	• Quality: Adherence to QA/QC protocols for sample preparation and data acquisition<br>• Boundary: Description of the potential impacts of methods (sample prep, chromatographic, MS) on observable chemical space<br>• Accuracy: Reported chromatographic and mass accuracy<br>• Precision: Variability of observed retention time, precursor mass error, and abundance	2	Chromatographic and mass accuracies/variability were not reported, but built into the workflow.
		Data Processing & Analysis QA/QC	• Quality: Outcomes of QC checks or filtering steps along the data processing & analysis workflow<br>• Boundary: Impact of data processing & subsequent analysis method(s) on observed chemical space, observed limits of detection/ID<br>• Accuracy: Performance measures (True Positive Rate, False Positive Rate, etc.) for known compounds or samples with known classification<br>• Precision: Reproducibility/repeatability of performance measures for known compounds or samples with known classification; Calculations such as False Discovery Rate, F1 score, etc.	2	Performance metrics described in great detail, limitations of database described.

Scoring System Explanation

Score	Explanation
0	No elements of relevant reporting are present.
1	Some elements of relevant reporting are present, but major improvements are needed.
2	Most elements of relevant reporting are present, but minor improvements are needed.
3	All elements of relevant reporting are present.
NA	Reporting not relevant to the study.
